# Parental beliefs and the influence of formal and informal literacy environments on preschoolers’ attitudes toward English learning

**DOI:** 10.1371/journal.pone.0329208

**Published:** 2025-08-06

**Authors:** Qingyun Li, Kimberley Kong, Qian Li, Qian Wang

**Affiliations:** 1 School of Educational Studies, Universiti Sains Malaysia, Penang, Malaysia; 2 School of Educational Studies, Chongqing College of International Business and Economics, Chongqing, China; 3 Institute for Advanced Studies, Universiti Malaya, Kuala Lumpur, Malaysia; National University of Malaysia Faculty of Education: Universiti Kebangsaan Malaysia Fakulti Pendidikan, MALAYSIA

## Abstract

English learning among Chinese preschoolers has increased in recent years. Despite policies that strongly oppose external English instruction for this age group, many parents are unrelenting and persist in providing English learning opportunities for their children. This study seeks to address how formal and informal literacy activities within the Home Literacy Environment (HLE) mediate the relationship between parental beliefs and Chinese children’s attitudes towards learning English in a foreign language context. Specifically, it explores: (1) how formal literacy activities within the HLE mediate this relationship, (2) how informal literacy activities within the HLE mediate this relationship. The study involved 405 participants who completed a questionnaire on family demographics, beliefs about the importance of early English education, the HLE, and children’s attitudes towards English learning. Quantitative data were analyzed using Structural Equation Modeling (SEM) with a Partial Least Squares (PLS) approach. Parental beliefs positively influence children’s attitudes towards English through formal and informal learning. These results highlight the importance of fostering a supportive and engaging HLE to enhance children’s positive attitudes towards learning English. The implications suggest that policy and practice should recognize the value of various literacy activities in early childhood education, particularly in the context of a foreign language and academically focused Asian society.

## Introduction

According to Chen et al. [1], research suggests that early bilingual education tends to foster positive attitudes towards target language learning and early immersion in a foreign language enhances cognitive development. This suggests the importance of early childhood for language acquisition [2]. Butler and Le [3] found that due to English’s status as an international lingua franca, many parents in non-English-speaking countries, particularly in East Asia, seek early English learning opportunities for their children. Given these reported benefits of early bilingual education, it is particularly important to examine how these principles apply in specific cultural and linguistic contexts, especially in countries like China where English serves as a foreign rather than second language and where unique policy and cultural factors shape the learning environment.

Attitudes play a crucial role in preschoolers’ English as a Foreign Language (EFL) learning. Children’s psychological readiness and propensity for language acquisition are shaped by their affective experiences, including preferences, intentions, and interests, which manifest in their active engagement and behavioral tendencies towards learning [4]. Kim [5] reported that children who are compelled to learn English from an early age may lose interest in the language. Children who did not enjoy learning tasks were found to have difficulty maintaining focus and interest, perceiving learning as challenging [5], implying that sustainable language learning relies on a genuine desire and enjoyment of the learning activities. Factors such as exposure to English, general language proficiency, personality traits, and attitudes towards English education all influence children’s English language acquisition [6], with attitudes being the most significant predictor of learning outcomes. Unlike adults, preschoolers typically acquire their foreign language implicitly rather than through explicit instruction [6–8]. In EFL contexts like China, where children learn English as a non-native language, the intrinsic motivation to socialize with peers in English is often absent, necessitating alternative strategies to enhance engagement [9].

During the preschool years, children’s homes serve as their primary learning environment. Given parents’ vital role during this period, understanding the factors that influence their EFL-related practices and decisions is essential. The home literacy environment (HLE) is where children are exposed to a range of literacy resources and interact with caregivers and it is widely recognized as a significant predictor of early literacy and language development for both monolingual and bilingual children [10]. Parental influences, such as quality interactions and a literacy-enriched home environment, are pivotal in developing language skills [11].

Importantly, the availability of home literacy activities and resources is influenced by parental beliefs (PB) on how much parents value education in general, and English learning in particular [12]. Parental beliefs (PB) refer to parents’ cognitive representations, values, and expectations regarding their children’s education and development, serving as cognitive frameworks that guide parental decision-making [12]. A recent experimental study [13] conducted among Chinese parents with limited English proficiency found that those with high expectations and positive beliefs about English learning were more proactive in assisting their children in learning the language. In a different study, it was found that Singaporean parents’ belief in having oral dialogues with children during shared reading positively predicted preschoolers’ reading interest [11].

However, despite compelling research evidence over the past three decades highlighting the significant impact of home environment on language and literacy skill development, a gap remains in understanding the specific experiential factors that contribute to individual differences in children’s learning attitudes. Research suggests that active parental involvement in learning activities, beyond just providing resources, plays a vital role in language development [14–16]. These early language skills tend to remain stable once formal learning begins [16]. It was also reported that without effective interventions, children who start behind their peers often remain behind [12]. Given the significance of attitudes in children’s long-term learning, it is imperative to identify the specific aspects of the HLE, whether physical resources or formal and informal approaches that foster optimal learning experiences. International research further supports the significance of HLE in language development [17]. Niklas et al. [18] demonstrated that HLE mediates the relationship between parental attitudes and children’s linguistic competencies, while Bingham [19] found that maternal literacy beliefs significantly influence mother-child reading interactions and early literacy development. Wirth et al. [20] revealed differential effects of HLE facets on children’s linguistic and socioemotional competencies, emphasizing its multidimensional nature. Recent research has focused on parental investment in English education within Asian contexts. Liu [21] examined parental investment and learners’ L2 Motivational Self System in China, revealing that parental economic, cultural, and social capital investments influence language learning success through a Bourdieusian capital perspective. Zhang et al. [22] found that parents’ strategic investments significantly predict students’ motivational orientations and learning outcomes, highlighting parental investment as a critical mediating mechanism between family resources and children’s language learning motivation.

Therefore, the current study seeks to investigate the effects of PB and the HLE on Chinese preschoolers’ attitudes towards EFL learning, providing deeper insights into parental practices in promoting early English education.

### English education in china and parents beliefs about EFL

Many Chinese parents appear to recognize the value of early English learning for their children’s future academic and career prospects [9]. According to Hu et al. [2], approximately one-third of Chinese parents enroll their preschoolers in language learning institutions for English education as of recent studies [2]. In Chinese cities such as Beijing, Shanghai, and Guangzhou, the focus on English learning, regardless of the learners’ age, is even more pronounced due to the cities’ global economic integration and the high demand for international communication skills [2,3]. This emphasis is further reinforced by the importance of English proficiency in key examinations such as the National College Entrance Examination (Gao Kao), International English Language Testing System (IELTS), and Test of English as a Foreign Language (TOEFL) [23], which are critical for accessing academic and professional opportunities.

The enthusiastic behavior of Chinese parents, who begin their children’s English learning early to provide them with educational advantages, attracted the attention of policymakers. Recognizing this trend, the Ministry of Education issued a policy prohibiting kindergartens from teaching English and excluding English lessons from the kindergarten curriculum in 2017 [24]. According to Hu [25], many language learning centres that cater to preschool-aged learners were also ordered to shut down. This policy aims to ensure that young children focus on holistic development and native language skills development, while also addressing broader concerns such as mastery of Mandarin, reducing academic pressure, ensuring educational equity, and preserving cultural heritage, as noted by Butler and Le [3]. Despite governmental restrictions, many parents turn to home-based learning to provide early English education for their children.

However, this shift towards home-based English learning presents several challenges, particularly regarding the learning environment for preschoolers. Parental practices and involvement, often shaped by their beliefs, play a crucial role in this setting. This environment directly influences preschoolers’ attitudes towards foreign language acquisition and content. Therefore, it is essential to examine the theoretical foundations that explain parent-child interactions within educational contexts.

Bronfenbrenner’s ecological theory provides insights into the significant role parents play in influencing the home environment to optimize a child’s learning [26]. According to this theory, a child’s development occurs within nested environmental systems that interact to influence learning outcomes. At the microsystem level, the family represents the most immediate and influential environment, where direct parent-child interactions, daily routines, and learning activities occur. The mesosystem level encompasses the connections between different microsystems, such as the relationship between home learning practices and formal educational settings. The macrosystem level includes broader cultural values, governmental policies, and societal attitudes toward education that shape family practices. Within this context, parents’ beliefs and practices are pivotal in shaping the home environment, as empirically supported by various global studies. These beliefs determine the type of support and resources provided at home, such as educational materials, learning spaces, and language-rich activities. The interaction between macrosystem policies and microsystem practices creates unique dynamics where parental investment in home-based English learning becomes not merely supplementary but essential for children’s early language exposure.

A study by Riches and Curdt-Christiansen [27] among Chinese families in Canada showed that parents who prioritize their children’s academic success invest in a variety of home literacy resources and organize weekly literacy activities. This trend is also observed among Chinese parents in China. Vasilyeva et al. [12] further emphasized that parental beliefs significantly influence the selection of educational materials and activities. For instance, parents who consider literacy skills crucial for preschoolers’ school readiness may allocate more time and resources to developing their children’s reading skills compared to those who prioritize socioemotional skills for young children.

Additionally, studies by Chen et al. [1] in China demonstrate that parents with developmentally appropriate beliefs about reading engage in more frequent reading sessions, maintain a greater supply of books at home and initiate reading activities earlier. This fosters higher-quality interactions during reading. In Korea, Choi et al.’s study [28] indicates that parents’ literacy beliefs indirectly influence the language and literacy outcomes of preschoolers through their provision of a supportive home literacy environment.

### Home literacy environment

There is no consensus on the concept of the Home Literacy Environment (HLE), which has become increasingly complex over time. Initially, HLE was linked to socioeconomic status (SES), with research indicating that children from lower socioeconomic backgrounds had poorer language skills and less interest in literacy [29]. Later, the focus shifted to specific literacy activities, such as shared book reading. Recent studies suggest that HLE is best viewed as a multifaceted concept encompassing various attitudes, activities, and resources. Different elements of HLE may relate to different aspects of children’s literacy and language skills [14,16]. According to Phillips and Lonigan [30], HLE includes (a) parents’ beliefs about literacy and their role in fostering their children’s literacy skills and (b) the actions parents take to support language and literacy development. In the context of the current study, HLE is operationalized as actions parents take to support literacy development that include a diverse array of literacy-related interactions and provision of resources, within the familial setting. In the current study, HLE is operationalized according to the distinction between formal and informal learning activities proposed by Sénéchal and LeFevre [16]. Formal learning activities were defined as structured, print-focused activities such as flashcard drills for vocabulary acquisition, alphabet and word writing exercises, enrollment in English certification courses (e.g., Cambridge Young Learners English tests), hiring personal English tutors, and participation in structured after-school English programs. In contrast, informal learning activities included meaning-focused, spontaneous interactions such as shared English book reading, singing English songs or rhymes, conversational practice in English, playing digital games with English content, and visiting libraries or bookstores together.

The conceptualization of HLE in the current study mirrors the Home Literacy Model proposed by Sénéchal and LeFevre [16] that makes a distinction between formal and informal learning activities. The formal learning activities focus on the printed text such as labeling letter names and recognizing letter sounds [16]. These activities centred on alphabet knowledge, word decoding, and phonological awareness are designed to build ‘inside-out’ domain skills essential for translating written text into spoken language and meaning [31]. In contrast, the informal learning activities focus on the meaning attached to print rather than print per se [16]. Informal learning activities, intended to build ‘outside-in’ domain skills [31], are usually less structured and more spontaneous to support the development of oral language, vocabulary, narrative understanding, and reading comprehension.

### Home literacy environment and children’s efl attitudes

Despite extensive research on the effects of HLE on first-language development, few studies have explored how HLE influences children’s attitudes towards learning a second language. The impacts of HLE on monolingual and bilingual learners may vary, and learning activities in a first language and a second language might serve distinct purposes. Therefore, conclusions drawn from first-language development studies should not be generalized to second-language development without supporting empirical evidence [32,33].

A review of the current literature reveals a dearth of studies on the association between HLE and children’s language learning attitudes. Most studies tend to focus on the effects of HLE on the development of children’s literacy skills, especially reading [11,34,35], rather than on children’s attitudes. Even less studies are available on attitudes of EFL young learners especially among those whose first language is linguistically very different from their second language. Hence, there is a knowledge gap that needs to be addressed about the relationship between HLE, and children’s EFL attitudes.

A study conducted in Singapore found that parent-child engagement in home learning activities such as writing and reading, is a strong predictor of a child’s interest in reading, even when controlling for age and parent’s education level [11]. Similar findings were echoed in a Chinese study [36], which found children showing higher levels of interest in language learning when parents engage shared in reading with them. In a more recent experimental study in China, findings showed that home-based literacy activities in low-SES Chinese families not only supported students’ English learning outcome and parents’ English vocabulary, but also promoted students’ motivation in English learning, and fostered parent-child relationship [13]. In this study, parents were given a series of take-home activities in which they were encouraged to interact and practice with their first-grade children in English speaking, listening, reading, and writing after school. These studies indicate that children may develop positive learning attitudes when their parents actively involve themselves in the learning process [31,36,37], rather than merely providing resources for children’s independent or outsourced learning. The positive socioemotional climate created by parent-child engagement may fosters greater interest in learning and makes language acquisition enjoyable [38], which are attributable to several factors. Parental involvement provides consistent exposure, reinforces learning, and builds confidence [37]. Interactive activities such as shared reading and storytelling create engaging experiences and foster a love for reading that provides rich and varied linguistic input. It can form the foundation for strong oral language development [31]. Additionally, parents’ positive attitudes towards literacy serve as role models, instilling similar values in their children [37]. Emotional support and encouragement from parents further enhance children’s motivation and interest in learning [31] though this may not contribute towards language skills development [11].

### Gaps in current literature

The theoretical necessity for this research emerges from the intersection of ecological systems theory and home literacy research in foreign language contexts. While Bronfenbrenner’s ecological systems theory [26] provides a framework for understanding environmental influences on child development, its application to foreign language learning in restrictive policy contexts remains underexplored. Specifically, the theory suggests that when macrosystem policies limit institutional language learning opportunities, microsystem processes (particularly parental beliefs and home practices) become more critical for development outcomes.

Furthermore, Sénéchal and LeFevre’s Home Literacy Model [16], while well-established for first language development, requires validation and potential modification for foreign language contexts where parents and children may have limited target language proficiency. The distinction between formal and informal learning activities may operate differently in EFL contexts compared to native language environments, particularly when cultural values emphasize structured learning approaches.

From a Bourdieusian capital perspective, parental investment in children’s English learning represents the conversion of family capital (economic, cultural, and social) into educational advantages [21,22]. However, the mechanisms through which these investments translate into children’s affective responses to language learning remain unclear. Understanding these processes is theoretically important for expanding ecological systems theory to encompass foreign language learning contexts and practically significant for supporting families navigating policy restrictions on early English education.

The association between parent beliefs, HLE and child attitudes towards learning English in a foreign-language context can be inherently more complex in the Asian societies. Scholars have suggested that Asian parents may hold a circumscribed or narrowly defined view of their responsibilities and activities in supporting their children’s learning [11]. In competitive Asian education systems, parents often choose formal approach to learning language or literacy learning [31]. They may prioritize teaching inside-out domain skills over engaging in verbal discussions, viewing the latter as a lost opportunity for instruction [11]. This emphasis on printed words over verbal exchange is also observed in Hong Kong, where English is learned as a foreign language [39]. Asian parents may be more likely to view parent–child reading as an investment rather than bonding [40]. For Chinese children, acquiring English is viewed as a valuable asset for the future in a globalized economy, where proficiency in English can open doors to greater educational and career opportunities. Additionally, Chinese parents who primarily speak their mother tongue might lack the proficiency and confidence in English to discuss learning content with their children. Consequently, verbal interactions may be less frequent and sophisticated in foreign-language contexts, as parents and children are less proficient in the language [33].

Hence, there is a need to clarify the inter-relationships between parental beliefs, specific type of literacy environment that affect children’s attitudes toward EFL learning. This present study is undertaken to explore how both formal learning activities and informal learning experiences uniquely contribute to children’s attitudes to learning English. By dissecting the distinct roles of these HLE components, targeted strategies can be developed for parents and educators to create optimally supportive and enriching environments that nurture positive learning attitudes in young learners.

### The present study

**Fig 1:**
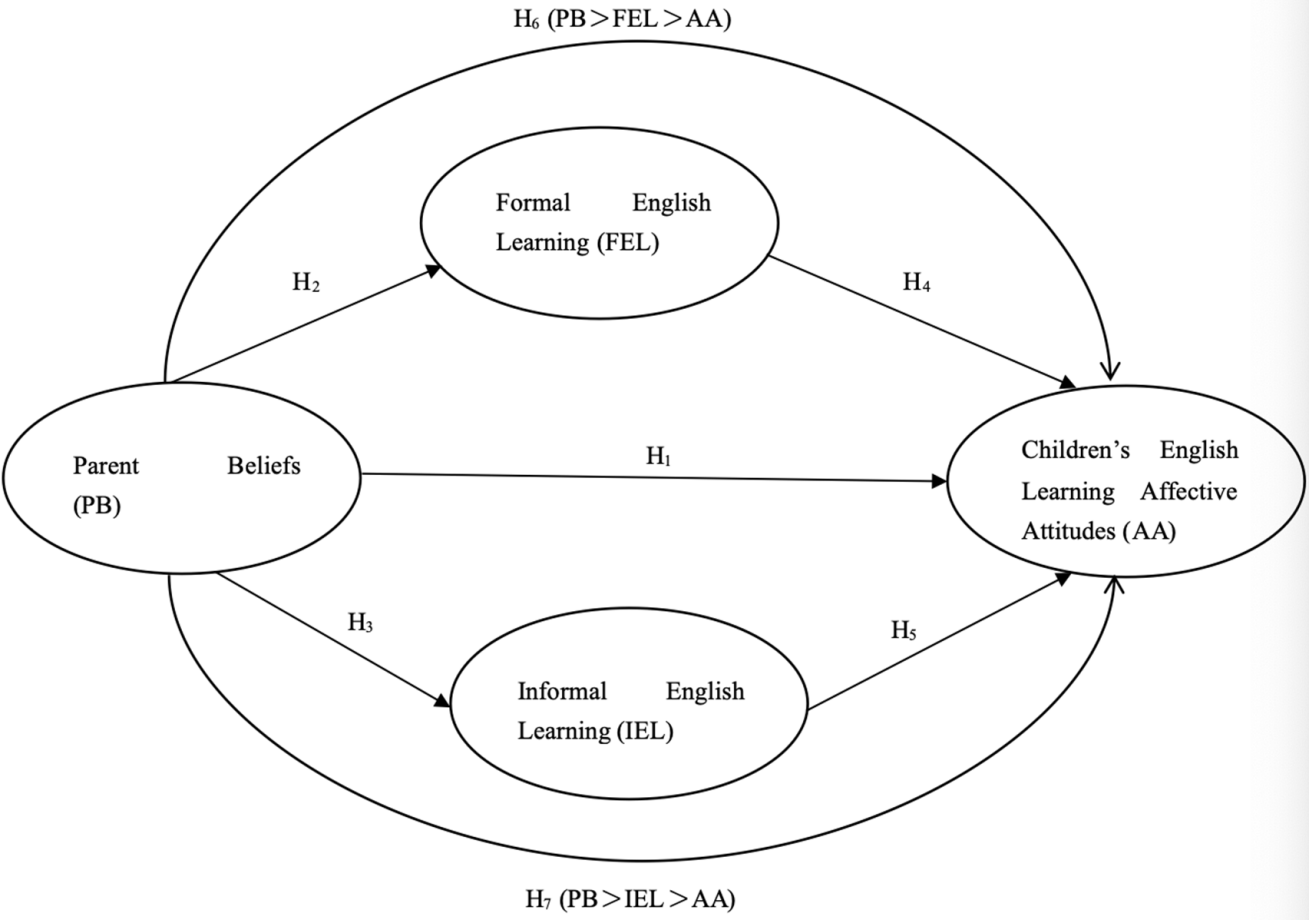
Conceptual framework. Note: Five direct relationship (H_1_ to H_5_) and two indirect relationships (H_6_ and H_7_).

The current study examines how parental beliefs (PB) and home literacy environment (HLE) predict preschoolers’ attitudes towards English learning. PB and HLE are conceptualized as antecedent variables that influence children’s attitudinal development rather than as components of the attitudes. Two research questions and conceptual framework (Fig 1) were proposed as follows.

1 How do parent beliefs, formal English learning, and informal English learning directly influence children’s affective attitudes towards English learning?

Hypothesis 1: Parent beliefs have positive effects on children’s affective attitudes towards English learning.

Hypothesis 2: Parent beliefs have positive effects on formal English learning.

Hypothesis 3: Parent beliefs have positive effects on informal English learning.

Hypothesis 4: Formal English learning has positive effects on children’s affective attitudes towards English learning.

Hypothesis 5: Informal English learning has positive effects on children’s affective attitudes towards English learning.

2 What is the nature of the indirect relationship between parent beliefs and children’s affective attitudes towards English learning mediated by formal English learning, and informal English learning?

Hypothesis 6: Formal English learning mediates the relationship between parent beliefs and children’s affective attitudes towards English learning.

Hypothesis 7: Informal English learning mediates the relationship between parent beliefs and children’s affective attitudes towards English learning.

## Methodology

The study used parent questionnaires to collect data on family background, home learning environment, and child attitudes.

### Study design and data collection

The study adopted a quantitative design using parent questionnaires to collect data on family background information, home learning environment, and children’s learning attitudes. Target participants were caregivers or parents of preschoolers from Zhengzhou, central China.

Data collection was conducted from July 7, 2024, to September 13, 2024, through Wenjuanxing (https://www.wjx.cn/), a Chinese online platform that provides paid services for researchers [41]. Participation calls were published on Chinese social media platforms including WeChat and Weibo. Eligible participants were parents of children aged 3–6 with at least 6 months home English learning experience.

### Participants

Using the Krejcie and Morgan [42] sample size determination guidelines, the recommended sample size for this study at a 95% confidence level with a 5% margin of error was calculated to be 385. The questionnaire was in Mandarin and designed to be self-explanatory. Participants completed the questionnaire in approximately 15 minutes. A small cash incentive of 5RMB was offered to each participant. Of 439 distributed questionnaires, 421 were returned within 7 days (96% response rate). Among returned questionnaires, 16 were incomplete, leaving 405 valid responses for analysis.

To enhance validity and reliability, the questionnaire included clear instructions, introductory messages, and detailed construct explanations [43]. Participants were assured anonymity and confidentiality [44]. Before data collection, two experts and six experienced parents reviewed the survey for content relevance and format. Since the survey was administered in China, bilingual versions were prepared with back-translation to ensure accuracy [45]. A pilot test of 60 responses from February 12 to March 1, 2024 validated questionnaire reliability with composite reliability exceeding 0.70 [46], indicating strong internal consistency of the items within the scales.

Respondents included 55.1% mothers (n = 223), 31.1% fathers (n = 126), and 13.8% guardians (n = 56). The majority of the caregivers (60.5%, n = 245) held at least an undergraduate degree. In terms of household income, 56.8% of participants reported annual earnings between 150,000–500,000 RMB. According to the Zhengzhou Statistics Bureau [47], the average annual income in Zhengzhou was 97,244 RMB in 2022, indicating that our participants predominantly represent middle-class families with above-average incomes.

### Ethics statement

This study followed ethical research standards. All participants provided informed consent. Since only parents completed questionnaires, no minors participated. Parents provided written consent after receiving detailed study information. Participation was voluntary with withdrawal rights. The study protocol was reviewed and approved by the ethics committee of the corresponding author’s university.

### Measures

The scales were selected based on three key criteria: (1) their established psychometric properties and validation in similar EFL contexts, particularly with Chinese populations; (2) the theoretical alignment with the study’s conceptual framework distinguishing formal and informal literacy activities as proposed by Sénéchal and LeFevre [16]; and (3) their ability to assess parental beliefs and children’s affective responses in cross-cultural language learning contexts. These instruments have been successfully adapted and used in previous research examining home literacy environments and language learning attitudes in Asian educational settings [9,28,48].

#### Parent beliefs.

The Parent Beliefs were measured using 11 items across two sub-dimensions: a) the value of early childhood English education (8 Items), b) the role of parents in their child’s English education (3 Items). The scale was adapted from a study by Lai et al. [9] and Zhang and Lau [48]. An example of the items is “*I believe that English learning should start early in my child’s education*”, “*I believe my interactions with my child during the process of English learning are very important”.* Each item is rated on a 5-point Likert-type scale (1 = strongly disagree, 2 = disagree, 3 = Uncertain, 4 = agree, 5 = strongly agree). Higher scores denote stronger parental beliefs in value of English learning and importance of their role to facilitate their children’s English learning. Cronbach’s alphas for the two dimensions were as follows: value of early childhood English education (> 0.90), the role of parents in their child’s English (> 0.80). The Parent Beliefs scale demonstrated satisfactory construct validity in this study. Confirmatory factor analysis revealed acceptable fit indices: χ²/df = 2.14, CFI = 0.96, TLI = 0.94, RMSEA = 0.053. Factor loadings ranged from 0.72 to 0.86, with all items loading significantly on their respective factors. Composite reliability was 0.92, and average variance extracted was 0.52, indicating adequate convergent validity.

#### Formal English learning.

Formal English Learning were measured using 7 items adapted from Forey et al. [49] and Lau and Richards [50]. It measures the amount of structured learning activities that are focused on the printed text that are designed to build skills essential for translating written text into spoken language and meaning [16]. Examples of the items include “*I carry out writing exercises with my child to help him learn alphabets and words*”, “*I use flashcards or drills to teach English words”.* Participants scored on a 5-point Likert-type scale (e.g., 1 = Never, 5 = Very often) to indicate their level of agreement with each statement. High score means parents provide more structured learning activities for their preschoolers’ English development. The Cronbach’s alpha values for the variables ranged from 0.80 to 1.00, satisfying the recommended criterion for internal reliability (e.g., 0.70). The Formal English Learning scale showed strong psychometric properties. Confirmatory factor analysis supported a unidimensional structure with good fit: χ²/df = 1.89, CFI = 0.97, TLI = 0.96, RMSEA = 0.047. All factor loadings exceeded 0.80, composite reliability was 0.93, and average variance extracted was 0.67, confirming the scale’s reliability and validity.

#### Informal English learning.

Informal English learning were measured using 7 items adapted from Lai et al. [9]. It measures less structured and more spontaneous learning activities focused on the meaning attached to print rather than print per se [16,31] to support the development of oral language, vocabulary, narrative understanding, and reading comprehension. Examples of the items includes “*I read books or look at picture books in English with my child”, “I sing English songs or rhymes to or with my child*”. To indicate how much they agreed with each statement, participants rated on a 5-point Likert-type scale (1 = Never, 2 = Rarely, 3 = Sometimes, 4 = Often, and 5 = Very often). A high score indicates that parents provide their preschoolers more informal English-learning activities. For the variables, the Cronbach’s alpha value was 0.89. The Informal English Learning scale demonstrated acceptable construct validity. Factor analysis revealed satisfactory fit indices: χ²/df = 2.03, CFI = 0.95, TLI = 0.93, RMSEA = 0.051. Factor loadings ranged from 0.72 to 0.82, composite reliability was 0.92, and average variance extracted was 0.62, supporting the scale’s psychometric adequacy.

### Children’s affective attitudes towards English learning

Children’s affective attitudes towards English learning were measured using 9 items adapted from Gardner’s Attitude/Motivation Test Battery [51]. These items measure children’s enjoyment, interest, motivation and preference in learning English. Examples of the items includes “*My child volunteers himself when asked to say something in English”, “My child prefers learning English to Chinese*”. Respondents rated items on a 5-point scale, where 1 = Strongly disagree, 2 = Disagree, 3 = Uncertain, 4 = Agree, and 5 = Strongly agree. Higher scores indicate more positive affective attitudes toward learning English, both informally and formally. The variables have a Cronbach’s alpha score of 0.92.

**Fig 2:**
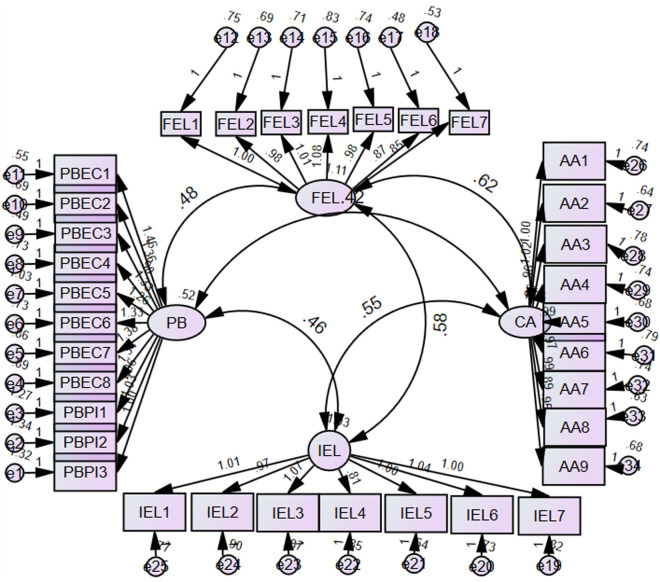
Measurement Model. Notes: PB - > Parent Belief; FEL - > Formal English Learning; IEL - > Informal English Learning; AA - > Affective Attitudes.

Table 1 presents the scale development. Confirmatory factor analysis (CFA) using SMART PLS was conducted to validate the factor structure. The model fitness values offer a comprehensive evaluation of the extent to which the model aligns with the data. The root mean square residual (RMR) value is 0.057, which falls below the recommended threshold of 0.08. This suggests that the model fits well, as shown by Hu and Bentler [52]. The Incremental Fit Index (IFI) is valued at 0.974. Values greater than 0.90 are believed to be suggestive of a strong correlation or agreement, according to Bentler and Bonett’s [53] research in 1980. The Tucker-Lewis Index (TLI) has a value of 0.972, which is above the recommended threshold of 0.90. This indicates a strong match, according to Tucker and Lewis [54]. The Comparative Fit Index (CFI) is valued at 0.974. Values greater than 0.95 are considered optimal, indicating a high level of agreement or compatibility [55]. The root mean square error of approximation (RMSEA) is a statistical measure used to assess a model’s goodness of fit. In this case, the RMSEA value is 0.026, which is lower than the recommended threshold of 0.06. This indicates good model fit [52]. The Chi-Square/Degrees of Freedom (CMIN/DF) ratio is 1.274, which falls below the threshold of 3.0. This suggests that the fit is good, as stated by Kline [56]. In general, the model fitness indices indicate that the CFA model fits the data well (refer Fig 2).

**Table 1:** Assessment of construct reliability and convergent validity.

	Construct	Loading
Parental belief about early childhood English education (PB) - α: 0.905, rho_A: 0.908, CR: 0.921, AVE: 0.518 & FC: 2.056		
PB 1:	I believe that English learning should start early in my child’s education	0.849
PB 2:	I believe that my child’s proficiency in English will give them a competitive advantage in the job market	0.800
PB 3:	I believe that English fluency will contribute to my child’s sense of identity and belonging in a globalized world	0.736
PB 4:	I believe that English learning enhances my child’s thinking and learning abilities	0.788
PB 5:	I believe that exposing my child to different cultures through learning English is important	0.721
PB 6:	I believe that knowing English helps my child communicate effectively with a diverse range of people	0.778
PB 7:	I believe that investing in my child’s English education is worth the financial resources required	0.774
PB 8:	I believe that learning English is crucial for my child’s overall growth and development	0.763
PB 9:	I believe I play an important role in my child’s English learning	0.862
PB 10:	I believe my interactions with my child during the process of English learning are very important	0.851
PB 11:	I believe in actively supporting and encouraging my child’s English learning by providing various activities and resources	0.833
Formal English Learning (FEL): - α: 0.916, rho_A: 0.917, CR: 0.933, AVE: 0.665 & FC: 2.247		
FEL 1:	Assist with English homework (online/private programs)	0.809
FEL 2:	Carry out writing exercises with my child to help him learn alphabets and words	0.815
FEL 3:	Use flashcards or drills to teach English words	0.819
FEL 4:	Hire a personal English tutor	0.816
FEL 5:	Enrol my child for English certification courses (e.g., IELTS, TOFEL, Cambridge Young Learners English (YLE) tests)	0.806
FEL 6:	Enrol my child for English enrichment after-school programs (e.g., English playgroups, tutorial classes)	0.829
FEL 7:	Travel with my children to take part in study tours	0.784
Informal English Learning (IEL): - α: 0.897, rho_A: 0.899, CR: 0.919, AVE: 0.619 & FC: 2.0123		
IEL 1:	Read books or look at picture books in English with my child	0.800
IEL 2:	Sing English songs or rhymes to or with my child, including bedtime songs	0.767
IEL 3:	Name objects or draw things with my child using English words	0.801
IEL 4:	Converse with my child in English	0.719
IEL 5:	Play digital games in English content with my child	0.816
ILE 6:	Visit bookstores or libraries with my child	0.811
ILE 7:	Develop an English study plan for the child	0.789
Children’s Affective Attitude (AA): - α: 0.926, rho_A: 0.927, CR: 0.938, AVE: 0.627 & FC: 2.187		
CA1:	My child enjoys learning English	0.795
CA2:	My child feels confident when asked to speak in English	0.817
CA3:	My child does not get anxious when he has to answer questions in English	0.775
CA4:	My child likes English learning activities; he looks forward to studying more English in the future	0.784
CA5:	My child volunteers himself when asked to say something in English	0.806
CA6:	My child prefers learning English to Chinese	0.776
CA7:	My child prefers learning English to Math	0.794
CA8:	My child feels bored during his English lessons	0.786
CA9:	My child feels disappointed when his English lessons/ learning activities are cancelled	0.792

Notes: a = Cronbach’s α; CR = composite reliability; AVE = average variance extracted;

FC = Full Collinearity

Additional demographic questions concerning respondents’ education, income, and their child’s age, gender, and English learning experiences were included (see Appendix A).

## Results

This study employed partial least squares structural equation modeling (PLS-SEM) to analyze the data. PLS-SEM is a multivariate statistical method used to examine relationships among multiple variables within a theoretical framework [57,58]. PLS-SEM is particularly suitable for prediction-oriented research goals, which aligns with the objective of understanding how parental beliefs predict children’s attitudes through mediating pathways [57,58]. PLS-SEM also demonstrates greater robustness to non-normal data distributions and smaller sample-to-parameter ratios, making it appropriate for complex models with multiple mediating relationships [59,60]. The variance-based approach of PLS-SEM is well-suited for exploratory research contexts where theoretical models build upon existing frameworks but examine novel relationships in under-researched populations [46]. Given its ability to handle complex structural models with higher-order constructs and multiple mediations [59,60], PLS-SEM (Smart PLS Version 4) was utilised to estimate the model parameters. First, a preliminary analysis was conducted to assess common method variance (CMV) and evaluate potential biases [61].

The variance inflation factor (VIF) values ranged between 2.06 and 2.19 well below the acceptable threshold of 3.3 [62]. Harman’s single-factor test revealed that the primary extracted factor accounted for only 27.06% of the total variance in the sample. This result, where both the individual and combined sample variances were notably below 40%, suggests that common method bias is unlikely to influence the findings [63].

Next, the data analysis proceeded in two stages: (i) assessment of the measurement model, ensuring the reliability and validity of the constructs, and (ii) assessment of the structural model, examining the relationships between the variables of interest [46].

### Assessment for reflective measurement model

Table 1 shows that the reflective measurement model achieved satisfactory convergent validity. Specifically, all average variance extracted (AVE) values exceeded the threshold of 0.5 [61,64], indicating that each construct explains more than 50% of its variance. Additionally, all indicator loadings surpassed the threshold of 0.7, and both composite reliability (CR), rho A, and Cronbach’s alpha values for each latent variable were above 0.7 [61,65,66], demonstrating strong reliability. Therefore, the measures demonstrated convergent validity.

Table 2 displays the discriminant validity of the measurement model. All HTMT values between construct pairs were below the 0.85 threshold [67], indicating that the constructs are sufficiently distinct from each other.

**Table 2:** Discriminant validity (HTMT_.85_) of measurement model.

	AA	FEL	IEL	PB
**AA**				
**FEL**	0.276			
**IEL**	0.200			
**PB**	0.540	0.589	0.590	

Notes: PB - > Parent Belief, FEL - > Formal English Learning, IEL - > Informal English Learning, AA - > Affective Attitude

### Assessment of structural model

To ensure the reliability of the path coefficients estimated by PLS-SEM, the study assessed potential collinearity issues among predictor constructs. As indicated in Table 3, the variance inflation factor (VIF) values were all below the critical threshold of 5 [68], confirming that collinearity did not bias the results in the structural model.

**Table 3:** Hypothesis Testing.

Path Hypothesis				Bootstrapped CI BC					
Variable Relationship	Path Coefficient Beta (β)	Standard Deviation (STDEV)	TStatistics (|O/STDEV|)	5% LL	95% UL	VIF	f^2^	R^2^	Q^2^predict
H_1_ PB - > AA	0.259	0.056	4..635**	0162	0.346	1.868	0.059	0.380	0.237
H_2_ PB - > FEL	0.589	0.036	16.296**	0.525	0.646	1.000	0.531	0.345	0.228
H_3_ PB - > IEL	0.590	0.032	18.706**	0.533	0.638	1.000	0.535	0.347	0.213
H_4_ FEL - > AA	0.276	0.053	5.235**	0.190	0.367	1.616	0.077		
H_5_ IEL - > AA	0.200	0.056	3.591**	0.103	0.289	1.620	0.040		
H_6_ PB - > FEL - > AA	0.163	0.035	4.715**	0.110	0.225				
H_7_ PB - > IEL - > AA	0.118	0.034	3.423**	0.060	0.175				

Notes: 1. This study used 95% confidence interval with a bootstrapping of 5,000; 2. *p < 0.05; **p < 0.01; 3. PB - > Parent Belief; FEL - > Formal English Learning; IEL - > Informal English Learning; AA - > Affective Attitudes; VIF (Variance Inflation Factor), Effect size (ƒ^2^), Coefficient of Determination (R^2^), Predictive Relevance (Q^2^)

Hypotheses were evaluated using bootstrap resampling with 5000 sub-samples (see Table 3). Parental beliefs about early childhood English learning had significant direct effects on children’s learning affective attitudes (β = 0.259, p < 0.01), of parent beliefs on formal English learning (β = 0.589, p < 0.01), and of parent beliefs on informal English learning (β = 0.590, p < 0.01), with 95% bias-corrected bootstrap confidence interval excluding zero (p < 0.001). Hence, H_1,_ H_2_ and H_3_ were supported.

Moreover, the direct relationship between formal English learning and children’s learning attitudes (β = 0.276, p < 0.01), informal English learning and children’s learning attitudes (β = 0.200, p < 0.01) were significant, with 95% bias-corrected bootstrap confidence interval without zero at the significance level of 0.05 and 0.001. Hence, H_4_ and H_5_ were supported.

**Fig 3:**
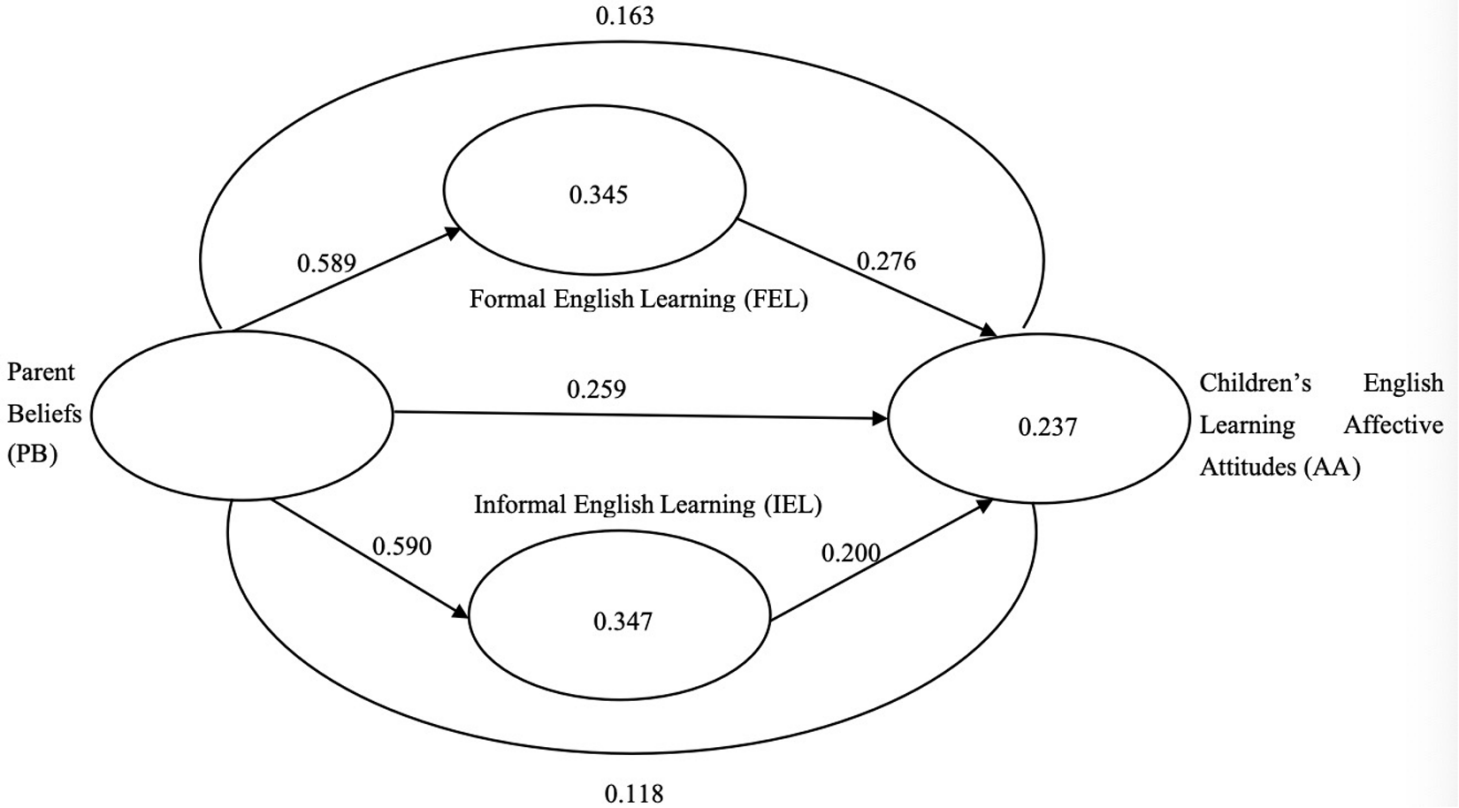
Structural model. Note: Index for lines are the standardized path coefficients (β) (e.g., parent beliefs > formal English learning, β = 0.589) and index for variables are the R^2^ values (e.g., R^2^ formal English learning = 0.345).

Further, there was a significant indirect effect of parent beliefs for early childhood English learning on children’s affective learning attitudes mediated by formal English learning environment (β = 0.163, p < 0.01), and informal English learning environment (β = 0.163, p < 0.01), with 95% bias-corrected bootstrap confidence interval without zero at a significance level of 0.05. Therefore, H_6_ and H_7_ were supported. Fig 3 illustrates the final structural model produced from the findings.

Additionally, to assess the quality of the structural model, coefficients of determination (R^2^), effect sizes (f^2^), and predictive relevance (Q^2^) were examined. The model explains 34.5% (Q^2^ = 0.228) of the variance in formal English learning, 34.7% (Q^2^ = 0.213) of the variance in informal English learning, and 38% (Q^2^ = 0.237) of the variance in children’s English learning affective attitudes, indicating a substantial explanatory power (see Table 3).

Effect sizes (f^2^) were analyzed to gauge the practical significance of each path. The path proposed in H_2_ and H_3_ demonstrated a large effect size (f^2^ = 0.531, 0.535), indicating its strong impact. Meanwhile, smaller but significant effect sizes were observed for paths in H_1_ (f^2^ = 0.059), H_4_ (f^2^ = 0.077) and H_5_ (f^2^ = 0.040) [46].

Finally, this study used PLS predict procedure to evaluate the predictive relevance of the model [69]. As shown in Table 3, the Q^2^ predict values for the endogenous constructs were greater than zero, indicating robust predictive relevance of the model. Building on this finding, we extended our prediction technique to estimate preschoolers’s attitudes towards EFL [70], demonstrating strong predictive validity (see Table 4).

**Table 4:** PLS-predict.

		PLS	LM	
Item	Q²_predict	RMSE	RMSE	PLS-LM
AA1	0.172	1.220	1.243	−0.023
AA2	0.179	1.196	1.218	−0.022
AA3	0.191	1.192	1.226	−0.034
AA4	0.182	1.186	1.199	−0.013
AA5	0.201	1.171	1.203	−0.032
AA6	0.149	1.231	1.264	−0.033
AA7	0.163	1.224	1.242	−0.018
AA8	0.190	1.093	1.115	−0.022
AA9	0.185	1.154	1.172	−0.018

Notes: LM stands for linear model; RMSE is for root mean square error. AA - > Affective Attitudes

Therefore, the proposed model demonstrates predictive validity in the target population, specifically regarding children’s attitudes towards EFL. This suggests the model may be useful for predicting English learning attitudes among preschoolers.

## Discussion

This study examined relationships among parental beliefs (PB), home literacy environment (HLE) and children’s attitudes towards learning EFL, with HLE as a mediator. The findings show that parental beliefs significantly influence children’s home literacy environment. Studies show that children whose parents value English education encounter more supportive learning environments [9,10,27], including formal English learning activities such as structured literacy instruction [49,71] and informal English learning through shared reading and storytelling [12,36]. This aligns with Bronfenbrenner’s ecological systems theory, in which parental beliefs function as proximal processes within the microsystem that directly influence child development outcomes [26]. This suggests that parental beliefs shape children’s attitudes towards English learning. Weigel et al. [37] found that mothers’ literacy beliefs related to home literacy environments and children’s literacy development. The importance of early positive attitudes is further supported by Gardner’s research [4] showing that affective factors serve as powerful predictors of second language learning success. When parents emphasise English education, children engage more actively in language studies [1,28].

The application of Bronfenbrenner’s ecological systems theory [26] to these findings reveals how different system levels interact to influence children’s English learning attitudes. At the microsystem level, the direct parent-child interactions during formal and informal learning activities create the immediate context for attitude development, where parental beliefs function as proximal processes that directly shape children’s experiences [26]. The mesosystem connections between home learning practices and children’s broader educational experiences help explain why formal activities may be more effective—they align with educational expectations children, creating consistency between home and anticipated school environments [26]. The macrosystem influence of Chinese cultural values emphasizing academic achievement [27,28] and the 2017 policy restricting kindergarten English instruction [24] creates the broader context that makes home learning environments critical for early English exposure, demonstrating how macrosystem factors can intensify the importance of microsystem processes when institutional pathways are restricted.

Additionally, the results from the first research question demonstrate that the home literacy environment during the early years significantly impacts the development of children’s attitudes towards English learning. This aligns with Silva et al. [72], who found that early English exposure creates positive responses in preschoolers’ emotional and attitudinal responses to learning English. However, formal learning activities demonstrated stronger associations with children’s positive attitudes (β = 0.276) compared to informal activities (β = 0.200). This challenges assumptions that play-based learning is more engaging for preschoolers [31]. In the Chinese context, formal activities like flashcards may provide clearer progress indicators and match cultural values that emphasize academic achievement [27,28]. This is notable since developmental theories emphasize the benefits of play-based learning for young children. Hypothesis 5 shows that informal English learning foster enthusiasm and positive connections to the language learning [73]. Choi et al. [28] also support this notion, suggesting that early informal English exploration enhances learning motivation and active participation among young learners.

These findings are significant in Chinese preschool English education. Strong parental belief influence on both formal and informal learning activities may reflects Chinese parents’ emphasis on educational achievement and willingness to invest in their children’s academic success [27]. This aligns with Vasilyeva et al. [12], who found that parental beliefs significantly influence educational material selection and activity organization. Riches and Curdt-Christiansen [27] found that academically-focused Chinese parents invest in a variety of home literacy resources and organize weekly literacy activities. These results are more significant given China’s 2017 policy banning kindergarten English instruction [24]. With formal institutional pathways restricted, home literacy environment becomes essential for early English exposure, making parental beliefs and practices the primary determinants of children’s early language learning experiences.

The mediation analysis revealed how parental beliefs influence children’s attitudes. Both formal (β = 0.163) and informal (β = 0.118) learning activities successfully mediated the relationship between parental beliefs and children’s attitudes, with stronger mediation through formal activities matching the direct effect. This suggests structured home-based learning compensates when institutional English learning pathways are restricted. This aligns with Choi et al. [28] who found that parents’ literacy beliefs indirectly influence preschoolers’ language and literacy outcomes through their provision of supportive home environments in Korea. This beliefs to practices to outcomes pathway supports the family investment model proposed by Vasilyeva et al. [12], where parental cognitive frameworks translate into concrete educational investments.

Finally, formal and informal home literacy activities mediated the relationship between parental beliefs and children’s attitudes. Parental beliefs positively influence children’s attitudes towards English through the informal interactions like storytelling and casual reading. The strong relationship (β = 0.259) suggests Chinese parents successfully create positive home learning environments despite lacking institutional support. This challenges concerns about home-based learning effectiveness, showing that strong parental beliefs can create effective learning environments. These results highlight the importance of fostering a supportive and engaging HLE to enhance children’s positive attitudes towards learning English. This aligns with research emphasizing the educational context’s role in shaping children’s language attitudes and the importance of supportive environments in mediating parental influences [1,74].

These findings are particularly important given China’s 2017 policy restricting English instruction in kindergartens [24]. The strong relationships between parental beliefs and home learning activities demonstrate that families are successfully compensating for institutional restrictions through private efforts. However, this compensation creates potential equity concerns, as families with stronger beliefs and greater resources are better positioned to provide rich learning environments.

The effectiveness of formal learning activities suggests that parents seeking to support their children’s English learning should consider incorporating structured elements alongside informal interactions. However, this must be balanced against developmental considerations emphasizing the importance of play-based learning for preschoolers.

In summary, HLE is a critical mechanism linking parental beliefs to children’ attitudes towards English learning.

## Conclusion

This study demonstrates that in the Chinese context, parental beliefs serve as powerful drivers of home literacy environments and children’s attitudes toward English learning. While both formal and informal learning activities mediate these relationships, formal activities show stronger associations with positive attitudes, challenging assumptions about optimal learning approaches for young children and highlighting the importance of cultural context in early language education.

### Implications, limitations, and future studies

This study examines how different home literacy approaches influence children’s attitudes towards learning English. Previous studies focused on how parental beliefs impact the home literacy environment. This study addresses overlooked families who integrate English education into daily life [2,23,50].

The findings can be interpreted through Bronfenbrenner’s ecological systems theory [26], which suggests that environmental systems influence child development. In the context of non-native English language acquisition, this framework helps explain how these systems interact. The findings highlight parents and the broader home environment as primary agents in either fostering or hindering early language development. The results show how parent beliefs significantly influence parental involvement in children’s education, thereby impacting their linguistic and cognitive development. Moreover, this study enhances Sénéchal’s Home Literacy Model [75], applying and testing its applicability in EFL contexts. It shows how different HLE aspects contribute to early English learning. The findings suggest this model needs to accommodate cultural variations in non-native language contexts.

The positive relationships suggest areas for educators and policymakers to consider. However, these should be interpreted cautiously given the cross-sectional design and the focus on middle-class families. Parental beliefs shape home learning environments, which relate to children’s attitudes. This suggests future intervention studies should explore supporting parental understanding of effective practices. However, longitudinal research is needed to establish sustained intervention effects. The stronger formal learning association should not be seen as endorsing structured over informal approaches. The optimal balance likely varies by child, culture, development, and family circumstances. Further research should examine how different approaches affect long-term motivation and development.

From a theoretical perspective, these findings show how home literacy environments function in foreign language contexts under policy restrictions. Future research could explore how different families navigate policy environments and whether these patterns generalize. For educational practice, the results suggest that supporting parental beliefs about English learning importance may be an effective intervention strategy. Parents should include structured elements while maintaining age-appropriate approaches. Educators should leverage parents’ formal learning investment while promoting informal interactions.

From a policy perspective, these findings suggest kindergarten English restrictions may burden families and worsen inequalities. Parents with stronger beliefs and resources can create richer environments, potentially disadvantaging others. Policymakers should consider supporting all families in creating positive learning environments.

Despite its implications, the study has limitations. It focuses on a specific cultural context and relies on self-reported data, which may affect generalizability and accuracy. The cross-sectional design limits causal interpretations of the relationships among PB, HLE, and children’s attitudes. Future research should use longitudinal designs to explore EFL motivation across cultures. Additionally, observing children and using multiple perspectives could provide detailed insights into learning motivation.

This study shows parental beliefs drive home literacy environments and children’s attitudes in China. While both formal and informal learning activities mediate these relationships, the stronger effects observed for formal activities highlight the importance of considering cultural contexts in early language education. The findings show family learning compensates for policy restrictions but requires balanced approaches for individual developmental needs.

## Supporting information

S1 Data Data Dictionary.
(XLSX)
